# Efficient Preparation of Ultrahigh-Strength Nanostructured Nickel by Ultranarrow Slit-Jet Scanning Electrodeposition Without Additives

**DOI:** 10.3390/mi17060700

**Published:** 2026-06-08

**Authors:** Zhenjian Lei, Pingmei Ming, Xinchao Li, Kun Wang, Wenjie Liu, Huan Liu, Shen Niu

**Affiliations:** 1 School of Mechanical and Power Engineering, Henan Polytechnic University, Jiaozuo 454003, China; 2 Guangdong HUST Industrial Technology Research Institute, Dongguan 523808, China; 3 School of Mechanical Engineering, Tongji University, Shanghai 201804, China

**Keywords:** nanostructured metal, ultranarrow slit-jet scanning electrodeposition, electrodeposition, ultrahigh-strength metal material

## Abstract

Electrodeposition of nanostructured metals often suffers from a trade-off between mechanical performance and efficiency. This study introduces ultranarrow slit-jet scanning electrodeposition (USJS-ECD), an additive-free technique employing a planar jet confined by a slit with opening width of <100 μm to scan the cathode. Numerical simulations coupling fluid flow and electric fields were conducted to optimize jet dynamics and scanning parameters. Experimental analyses reveal that USJS-ECD creates a highly localized, uniformly intensified energy field enabling direct fabrication of ultrahigh-strength nickel. The resulting deposits exhibit 98.82 wt% purity, an ultrafine grain size of 21.86 nm, and a mirror finish with surface roughness (Ra) of ~22 nm. Mechanical testing demonstrates a microhardness of 623 HV, a tensile strength of 756 MPa, and an elongation of 9.33%, achieving a superior strength-ductility synergy. Crucially, the deposition rate reaches 1.72 μm/min, significantly outperforming advanced ultrafine anode scanning electrodeposition (UAS-ECD) techniques. USJS-ECD presents a promising, efficient methodology for producing high-performance nanocrystalline metallic materials.

## 1. Introduction

Nanocrystalline metallic materials, defined by grain sizes on the nanometer scale, typically demonstrate enhanced overall mechanical properties, such as elevated strength, increased hardness, superior wear resistance, and potentially high toughness [1,2,3]. Moreover, these materials exhibit a high level of microstructural uniformity and functional adaptability, indicating significant potential for applications in aerospace, precision manufacturing, microelectronic devices, and functional coatings [4,5]. Nonetheless, despite the ability to achieve scalable production and superior material performance, the development of efficient, cost-effective, and large-scale fabrication methods remains a critical challenge for the practical engineering application of nanocrystalline metallic materials [6].

Among the various fabrication methodologies, electrodeposition is widely recognized as an effective technique for synthesizing nanocrystalline metals and their coatings. This is attributed to its mild processing conditions, minimal equipment requirements, high material utilization efficiency, and excellent compatibility with substrates exhibiting complex geometries [7,8,9]. Substantial research efforts have been directed toward advancing conventional electrodeposition strategies for nanocrystalline metals, including pulse current electrodeposition [10], high overpotential deposition [11], and the addition of organic or sulfur- and nitrogen-containing additives to the electrolyte [12]. Although these approaches demonstrate considerable grain refinement capabilities, they present notable limitations in practical applications. Specifically, pulse current electrodeposition primarily depends on transient high overpotentials to enhance nucleation, with its regulatory effectiveness contingent upon the periodic recovery of the diffusion layer; this dependence complicates its maintenance under conditions of high deposition rates or increasing deposit thickness [13,14]. High overpotential deposition can lower the nucleation energy barrier but is typically accompanied by vigorous hydrogen evolution and local pH fluctuations, which readily induce various defects [15,16]. Grain size control via additives inevitably introduces impurity elements such as sulfur, carbon, and nitrogen, which tend to segregate at grain boundaries, thereby compromising the intrinsic ductility, thermal stability, and long-term service reliability of the materials. This issue is particularly pronounced in high stacking fault energy metals such as nickel, magnesium, and aluminum, where impurity segregation can significantly modify stacking fault energy, suppressing strengthening mechanisms related to twinning or stacking faults and thus hindering the simultaneous attainment of high purity and grain refinement [17]. Consequently, conventional electrodeposition techniques generally encounter a trade-off among achieving high strength, hardness, and toughness, which substantially limits the engineering applications of nanocrystalline metals [18].

In this context, there has been growing interest in the controlled synthesis of nanocrystalline nickel under additive-free conditions by manipulating the physical environment at the deposition interface [19,20]. Zhu et al. [21] introduced an innovative mechanical electrodeposition method wherein dynamic hard particles are incorporated during deposition to continuously polish the cathode surface and disrupt the solution environment near the interface. This additive-free process yielded nickel deposits with refined grain sizes ranging from 30 to 80 nm, accompanied by bright, smooth surfaces and a marked reduction in defects such as pores, pinholes, and nodules. The microhardness of the deposits was significantly enhanced, demonstrating that physical disturbances at the interface alone can profoundly affect nucleation behavior and grain growth mechanisms.

Building upon this foundation, Liu et al. [22] developed a controllable friction-assisted jet electrodeposition technique, employing a rolling nozzle to apply continuous and regulated pressure during deposition. This approach effectively inhibited the rapid growth of coarse grains. Under optimized conditions, the limiting current density for nickel coating fabrication increased to 200 A/dm^2^, representing 1.54 and 1.43 times the values achieved by conventional jet electrodeposition and flexible friction-assisted jet electrodeposition, respectively. This method substantially improved deposition efficiency while preserving high surface quality. The combined effects of physical disturbance and jet-enhanced mass transfer were instrumental in overcoming stability limitations during high current density deposition.

Beyond interfacial disturbance, the influence of mass transfer conditions and electric field distribution on nucleation behavior has been extensively studied. Zhang et al. [23] designed a three-electrode system incorporating fluid injection to systematically examine the nucleation and growth dynamics of Ni^2+^ ions under conventional, vertical, and jet electrodeposition conditions. Their findings revealed that jet-induced convection significantly modifies interfacial mass transport and charge distribution, thereby regulating nucleation modes and growth kinetics of nanoclusters. At an early deposition stage of 0.5 s, nucleation particles exhibited diameters predominantly between 10 and 11 nm, which increased to 19–21 nm at 2 s, indicating that jet electrodeposition facilitates the most rapid nucleation process.

In a related development, Xiao et al. [24] proposed an ultrafine anode scanning electrodeposition (UAS-ECD) technique, where in an ultrafine, electrochemically inert anode with a characteristic dimension of approximately 50 μm is reciprocally scanned within an extremely narrow interelectrode gap of about 200 μm. Under additive-free conditions, this method successfully produces nanostructured nickel with a purity of up to 99.9997%. The resulting nickel exhibits outstanding mechanical properties, including a microhardness of 656 HV, an ultimate tensile strength of approximately 664 MPa, and an elongation to failure of 8.5%, demonstrating that a highly localized electric field enables the concurrent attainment of high strength, purity, and ductility. This approach offers a novel and promising pathway for the large-area fabrication of high-performance nanocrystalline metallic materials.

However, the use of an inert anode entails continuous consumption of metal ions from the primary electrolyte salt, which is unavoidable. Moreover, insufficient mass transport poses challenges in maintaining consistent metal deposition properties and stable deposition rates. If the issues related to limited deposition efficiency and fluctuations in electrolyte composition are effectively addressed, while preserving the intrinsic benefits of this technique—such as large-area single-step fabrication, facile real-time electric field modulation, and high material purity—its applicability in a wider range of engineering contexts can be significantly enhanced.

To address the limitations inherent in conventional scanning electrodeposition techniques that utilize inert ultrafine anodes, a novel approach termed ultranarrow slit-jet scanning electrodeposition (USJS-ECD) is introduced. Distinct from methods that localize the electric field through the direct application of ultrafine linear inert anodes, USJS-ECD employs a macroscale soluble anode in place of the microscale inert anode, thereby maintaining a relatively stable concentration of metal ions within the electrolyte. Concurrently, a localized electric field is established by channeling the electric field generated by the soluble anode through a microscale slit, in conjunction with a high-velocity planar jet. This configuration aims to overcome the challenges of limited mass transport efficiency and difficulties in product removal commonly associated with scanning electrodeposition processes based on ultrafine inert anodes.

During the USJS-ECD process, the planar jet continuously enhances forced convection at the cathode interface throughout scanning, effectively mitigating concentration polarization and substantially improving the interfacial replenishment of Ni^2+^ ions. Additionally, the scanning motion induces periodic migration of regions exhibiting high current density across the deposition area, thereby preventing prolonged localization of current that could otherwise result in inadequate mass supply, defect accumulation, and nonuniform thickness or microstructural features. In comparison to traditional scanning electrodeposition methods that rely exclusively on the motion of ultrafine inert anodes, USJS-ECD introduces a synergistic coupling between convective enhancement and localized electric field effects, supplemented by the use of a soluble anode. This synergy facilitates the formation of nanocrystalline structures characterized by both high nucleation density and an accelerated formation rate under stable conditions.

Through extensive numerical simulations and experimental analyses, the interrelated dynamics of fluid flow and electric fields during USJS-ECD have been systematically examined. The interactive influences of critical parameters—such as slit width, scanning velocity, interelectrode voltage and planar jet velocity—on microstructural development, deposition rate and the mechanical properties of nickel coatings have been elucidated. The results indicate that USJS-ECD not only retains the benefits of ultrafine inert anode scanning electrodeposition in producing high-purity nanocrystalline nickel without additives but also significantly surpasses conventional UAS-ECD in terms of deposition efficiency, structural homogeneity, and the simultaneous enhancement of hardness and toughness.

## 2. Process Scheme of USJS-ECD

### 2.1. Working Principle of USJS-ECD

**Figure 1:**
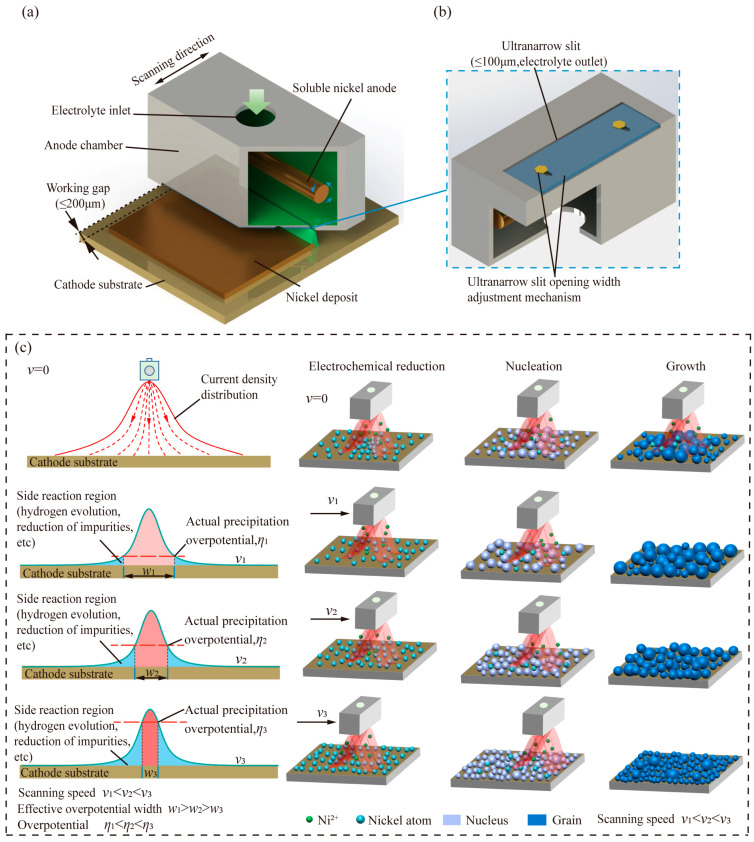
Working scheme and mechanism of USJS-ECD. (**a**,**b**) schematic diagrams of USJS-ECD; (**c**) overpotential distribution changing with the scanning speed and schematic diagrams of grain size distribution under the varying scanning speed.

The configuration of the ultranarrow slit jet scanning electrodeposition (USJS-ECD) system is illustrated in Figure 1a, featuring an overall dynamic scanning architecture. The anode chamber is positioned above the planar cathode, and the gap between the slit outlet and the cathode surface is precisely maintained at approximately 200 μm by a high-precision displacement and gap control system. During deposition, the anode chamber performs reciprocating linear motion along the cathode surface, resulting in periodic migration of the electrochemically active region across the interface. As shown in Figure 1b, when the applied potential drives the current through the ultranarrow slit with an adjustable opening width, the electric field is spatially confined and redistributed, resulting in a Gaussian-like distribution, as illustrated in Figure 1c. The slit opening width is smaller than 100 μm, while its length can be adjusted according to the desired deposition area. This geometric confinement induces a highly concentrated electric field and reaction current along the transverse direction, while extending along the slit length, thereby forming a strip-shaped high-activity deposition region on the cathode surface. The effective lateral extent of this region is strictly governed by the slit dimensions, enabling geometric control over the spatial distribution of the electric field and mass transport. Meanwhile, within the anode chamber, the active nickel rods are driven to rotate synchronously by a motor through a magnetic timing belt system. This mechanism ensures uniform anodic dissolution and a continuous supply of nickel ions, thereby enhancing the stability of both the electric field and the overall deposition process.

The formation mechanism of nanocrystalline nickel in the USJS-ECD process is similar to that in the UAS-ECD system, as illustrated in Figure 1c. With increasing scanning speed, the residence time of the jet at a given location is significantly reduced, leading to a progressively narrowed and intensified overpotential distribution. As a result, the reduction rate of nickel ions increases within a unit time, giving rise to a higher concentration of nickel atoms on the cathode surface and accelerating the formation of atomic clusters. Once these clusters exceed the critical size, they transform into stable nuclei.

At the same time, the reduction in the effective overpotential region compresses the spatial area involved in deposition. Due to the rapid scanning motion, the system moves away from a given location shortly after stable nuclei are formed, and no longer provides sufficient energy or reactant supply for further growth. Consequently, the nuclei do not have adequate time to grow into larger grains, resulting in the formation of nanocrystalline structures.

Through the integrated design of slit confinement, scanning modulation and dynamic anodic dissolution, the USJS-ECD system enables precise control over the deposition region, electric field distribution and interfacial mass transport conditions. This configuration provides a robust experimental platform for investigating deposition behavior and microstructural evolution.

### 2.2. Multiphysics Numerical Simulation of USJS-ECD

#### 2.2.1. Models

To understand the localized electrohydrodynamic characteristics of the USJS-ECD process, numerical simulations were carried out using COMSOL Multiphysics 6.0. The primary objective of the simulation was to qualitatively investigate the coupling behavior between the flow field and electric field under representative slit-jet-assisted conditions, as well as the localized deposition tendency under stationary deposition conditions. Since the actual USJS-ECD process involves continuously evolving reciprocating scanning motion and long-term cumulative deposition behavior, direct simulation of the complete dynamic scanning process would require considerably more complex moving-boundary calculations and transient interface tracking. Therefore, the present study adopts a representative quasi-steady-state stationary slit-jet model to analyze the localized flow-electric coupling characteristics beneath the slit region.

**Figure 2:**
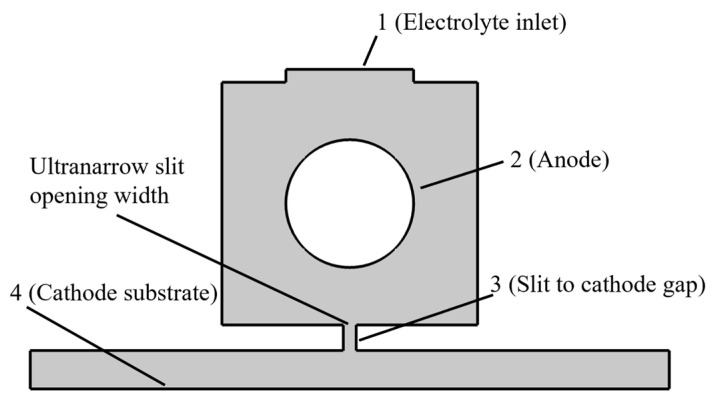
Physical model and boundary conditions of the USJS-ECD.

Figure 2 illustrates the physical model and corresponding boundary conditions employed in the simulation. During the deposition process, electrolyte is continuously ejected through an ultranarrow slit opening and impinges onto the cathode surface, thereby generating localized convection-enhanced mass transfer and electric-field concentration within the deposition region. Considering the geometric symmetry of the slit structure and the primary focus on localized interfacial behavior beneath the slit region, the two-dimensional cross-sectional model was established to simplify the numerical calculation while retaining the dominant electrohydrodynamic characteristics of the process.

To simplify the numerical calculation without losing generality, the following assumptions were adopted:

(1) The electrolyte was treated as a continuous incompressible Newtonian fluid under laminar-flow conditions.
(2) Representative stationary slit-jet conditions under quasi-steady-state assumptions were employed instead of fully dynamic reciprocating scanning conditions.
(3) The cathodic reaction was assumed to consist solely of nickel electrodeposition, while cathodic side reactions such as hydrogen evolution were neglected.
(4) Thermal effects, gas evolution, and moving-boundary deformation during long-term deposition were neglected.
(5) The simulation mainly focused on localized current-density distribution and mass-transfer behavior rather than exact quantitative prediction of macroscopic deposition thickness.

Because the electrodeposition process involves coupled fluid flow, ion transport, and electrochemical reactions, corresponding governing equations were introduced to solve the simplified model. The flow behavior of the electrolyte was governed by the incompressible Navier-Stokes equations:

(1) $$\rho \left(\frac{\partial u}{\partial t}+u\cdot \nabla u\right)=-\nabla p+\mu \nabla^{2}u$$

The electrochemical interfacial behavior was described using the secondary current distribution model:

(2) $$\nabla \cdot \left(\sigma \nabla \phi \right)=0$$

The transport behavior of Ni^2+^ ions within the electrolyte was governed by the Nernst-Planck equation:

(3) $$\frac{\partial c_{i}}{\partial t}=\nabla \cdot N_{i}=R_{i}$$

(4) $$N_{i}=-D_{i}\nabla c_{i}+z_{i}u_{i}c_{i}\nabla \phi +uc_{i}$$

The electrolyte consists of nickel sulfamate system and the remaining simulation parameters are listed in Table 1.

#### 2.2.2. Simulation Results and Discussion

(1) Distributions of flow field and current density

**Figure 3:**
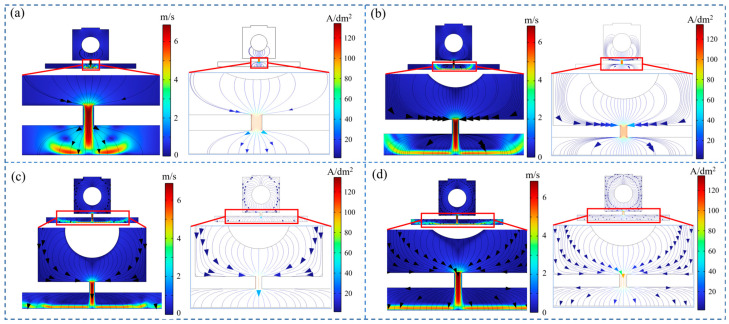
Simulated outcomes depicting the interaction between the electric and flow fields during the progression from electrolyte ejection to complete coverage of the cathode surface, alongside the distribution of the electric field independently. (**a**) t = 0.5 s, representing the initial phase; (**b**) t = 1 s, with the electrolyte covering approximately half of the cathode surface; (**c**) t = 1.5 s, indicating the electrolyte has just fully covered the cathode surface; (**d**) t = 2 s, corresponding to the jet attaining a stable laminar flow state.

Coupled interaction between the flow field and the electric field, together with independently simulated electric field distribution, is shown in Figure 3. Over a period of 2 s, the electrolyte propelled by a planar jet progressively disperses across the cathode surface, during which the electric field distribution transitions from a localized concentration to a more globally uniform pattern.

At the initial stage (Figure 3a), a distinct zone of concentrated electric field emerges in the area corresponding to the slit. As the electrolyte coverage expands (Figure 3b,c), the electric field gradually diffuses outward, becoming increasingly homogeneous. Upon reaching a steady state (Figure 3d), a coupling between the flow and electric fields is established, yielding a relatively uniform secondary current distribution across the cathode surface and creating a highly homogeneous electrohydrodynamic interaction region. Within this coupled spatial domain, the local current density can be approximated by Equation (5).

(5) $$j=nFD\frac{c_{bulk}-c_{surface}}{\delta }$$

where *j* denotes the current density, *c_bulk_* and *c_surface_* represent the bulk concentration and the electrode surface concentration, respectively, *F* is the Faraday constant, *n* is the number of transferred electrons and *D* is the diffusion coefficient, *δ* is diffusion layer thickness.

As illustrated in Figure 3a–d, the current density profile demonstrates a spatial distribution characterized by convergence at the central region, followed by a gradual radial extension, closely resembling a Gaussian distribution. Theoretically, the peak current density at the slit center attains a value of approximately 125 A/dm^2^, subsequently diminishing progressively along the cathode surface toward both lateral edges without exhibiting any discernible discontinuities. Concurrently, the electrolyte, propelled by a theoretical jet velocity of approximately 6 m/s, continuously impinges upon the cathode surface, thereby alleviating concentration polarization and enhancing the convective transport of Ni^2+^. This process substantially augments the interfacial mass transfer efficiency.

(2) Formation mechanism of nanocrystalline nickel and localized stationary deposition behavior

**Figure 4:**
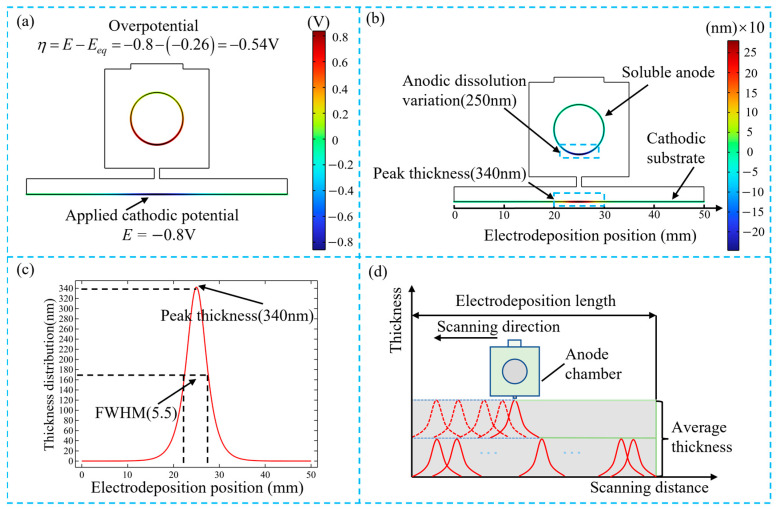
(**a**) Formation conditions of nanocrystalline; (**b**) evolution of anodic dissolution and cathodic deposition at t = 10 s with fixed anode chamber; (**c**) corresponding theoretical deposition thickness profile on the cathode surface at t = 10 s with fixed anode chamber; (**d**) formation mechanism of the nickel deposit.

The synthesis of nanocrystalline nickel through USJS-ECD is primarily influenced by the establishment of an effective overpotential (*η*). The magnitude of this overpotential, when maintained within an optimal range, is crucial for facilitating the process of nanocrystallization. This overpotential is characterized as the difference between the actual electrode potential and the equilibrium potential:

(6) $$\eta =E-E_{eq}$$

(7) $$E_{eq}=E^{0}+0.0139\ln\left(0.42c\right)$$

where *E* denotes the actual cathodic potential of the working electrode and *E_eq_* represents the thermodynamic equilibrium potential of the electrode reaction under given conditions. *E*^0^ corresponds to the standard electrode potential of Ni^2+^/Ni with a value of −0.25 V and *c* denotes the concentration of Ni^2+^. The overpotential varies dynamically during practical electrodeposition, while the present study employs a characteristic value obtained under quasi steady conditions from numerical simulation, with actual values fluctuating around this range. Nickel sulfamate electrolyte with a concentration of 350 g/L is adopted, corresponding to Ni^2+^ concentration of approximately 1.4 mol/L. As shown in Figure 4a, the theoretical cathodic potential *E* is −0.8 V. Substitution of these parameters into Equations (6) and (7) yields an overpotential *η* of approximately −0.56 V. This value exhibits a pronounced deviation relative to the equilibrium potential *E_eq_* of −0.26 V, indicating that the system operates under a high overpotential deposition conditions, which facilitates nucleation while suppressing grain growth, thereby satisfying fundamental conditions for formation of nanocrystalline structures.

**Figure 5:**
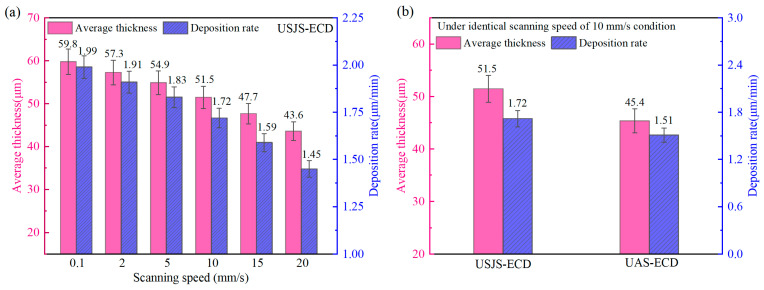
(**a**) Thickness and deposition rate of nickel deposits fabricated by USJS-ECD at different scanning speeds for 30 min; (**b**) comparison of thickness and deposition rate of nickel deposits prepared by USJS-ECD and UAS-ECD [24] at the same scanning speed of 10 mm/s for 30 min.

Under the aforementioned multiphysics coupling conditions, after 10 s of stationary deposition, significant material dissolution is observed at the anode, characterized by a corrosion peak of approximately 250 nm. Concurrently, a nickel deposit forms on the cathode surface, reaching a maximum thickness of about 340 nm (theoretically calculated vertical deposition rate of 34 nm/s). The lateral thickness profile of the deposit displays a characteristic approximately Gaussian distribution, with a theoretical full width at half maximum (FWHM) of approximately 5.5, as illustrated in Figure 4b,c. The observed asymmetry between the anodic dissolution peak and the cathodic deposition peak suggests that mass transport within the system results from the combined influence of continuous dissolution from the active nickel anode and convection-enhanced transport induced by the planar jet. Building on this, the localized high deposition rate, coupled with reciprocating linear scanning motion depicted in Figure 4d, facilitates the rapid formation of a nanocrystalline nickel layer. This layer consists of a continuous tiling and superposition of numerous microscale or nanoscale Gaussian-shaped deposition units distributed over a two-dimensional plane within a relatively short time frame. Further comparison of deposition thickness and rate between USJS-ECD and UAS-ECD, as presented in Figure 5, corroborates this phenomenon, with USJS-ECD demonstrating a significantly enhanced deposition rate relative to UAS-ECD.

## 3. Experimental

### 3.1. Sample Preparation

In this investigation, a nickel sulfamate electrolyte system was utilized as the deposition medium. To mitigate anodic passivation during the deposition process, an appropriate concentration of NiCl_2_ was incorporated into the electrolyte, compensating for the use of an active anode. To preserve the high purity of the deposited nickel layer, no organic or inorganic brighteners or grain-refining additives were introduced. The electrolyte composition comprised 350 g/L Ni(NH_2_SO_3_)_2_·4H_2_O, 5 g/L NiCl_2_, and 20 g/LH_3_BO_3_ all of analytical grade with a minimum purity of 98%, procured from Macklin, Shanghai. The electrolyte temperature was rigorously maintained at 50 ± 1 °C via a thermostatic control system. The pH was adjusted and stabilized at 4.0 ± 0.1 through the addition of basic nickel carbonate. An online monitoring and replenishment system was employed to ensure that the concentrations of nickel ions and boric acid remained within specified limits throughout the deposition process.

The anode consisted of a high-purity nickel rod (≥99.9% purity) with a diameter of 10 mm and length of 100 mm. It was secured within the anode chamber using a magnetic fixture and driven by a permanent magnet synchronous pulley located at the chamber’s left end to facilitate stable rotation. This configuration ensured continuous anodic dissolution and maintained electric field stability. The cathode substrate was a SUS304 stainless steel plate measuring 60 mm × 60 mm × 2 mm, with an effective deposition area of 50 mm × 50 mm; non-deposition regions were electrically insulated.

During electrodeposition, a constant voltage of 20 V was applied. The slit opening width was set to 50 μm, with its length corresponding to the effective deposition area. The working gap between the slit and the cathode surface was maintained at 200 μm, and the electrolyte flow rate supplied to the anode chamber was controlled at 16 L/min. To examine the influence of scanning velocity on deposition behavior, six scanning speeds of 0.1 mm/s, 2 mm/s, 5 mm/s, 10 mm/s, 15 mm/s and 20 mm/s were selected for comparative analysis. After electrodeposition, the deposited nickel foils were carefully separated from the SUS304 substrate by mechanical peeling using a thin blade for subsequent characterization and mechanical testing.

For subsequent microstructural characterization, mechanical property assessment, and related analyses, self-supported nickel foils with thicknesses ranging from 40 to 65 μm were fabricated using the USJS-ECD process. Additionally, previously reported data obtained via the UAS-ECD method were employed as a reference benchmark to facilitate comparative evaluation and mechanistic interpretation of deposition behavior, microstructural evolution, and mechanical properties, thereby ensuring consistency in physical parameters and processing conditions across different fabrication techniques.

### 3.2. Methods for Microstructural Characterization and Mechanical Property Evaluation

Surface morphology and cross-sectional microstructure of the deposited layers were examined via scanning electron microscopy (SEM) using a HITACHI SU-8010 (Tokyo, Japan) field emission SEM operated at an accelerating voltage of 20 kV. Crystallographic analyses were conducted employing a JEOL 2100 (Tokyo, Japan) transmission electron microscope (TEM) and a Bruker D2 X-ray diffractometer (XRD) (Bruker, Karlsruhe, Germany). Surface roughness measurements were performed utilizing an Olympus IX83-FV3000 (Tokyo, Japan) confocal laser scanning microscope. Elemental composition, including metallic and impurity contents, was quantified through inductively coupled plasma mass spectrometry (ICP-MS) using an Agilent 7500 system (Santa Clara, CA, USA), complemented by analysis with a FLASH2000 organic elemental analyzer (Thermo Fisher Scientific, Milan, Italy). Microhardness testing of both as-deposited and annealed nickel samples was carried out using a JITAI KEY HV1000 (Beijing, China)Vickers hardness tester under a load of 100 g with a dwell time of 15 s. The annealing treatment was carried out under vacuum at 200 °C for 4 h using a SA2-6-12TP DROIDE (Suzhou, China) vacuum heating furnace. For specimens exceeding 50 µm in thickness, a minimum of five hardness measurements were taken at different locations and averaged to enhance data reliability. Tensile specimens were machined into dumbbell shapes with a gauge length of 30 mm and a width of 6 mm; thickness was precisely measured using a Mitutoyo 547–301 digital thickness (Kawasaki, Japan) gauge with a resolution of 0.1 µm. Uniaxial tensile tests were performed on a DANA DNS5000N (Jinan, China)electronic universal testing machine at a constant strain rate of 2 mm/min, each sample was subjected to no fewer than two tensile tests.

## 4. Results and Discussion

### 4.1. Deposition Rate of Nickel Samples

The variation in thickness and the corresponding deposition rate of nickel samples prepared by USJS-ECD at different scanning speeds is shown in Figure 5a. To quantitatively assess the deposition characteristics, the deposition rate is defined as the change in deposit thickness per unit time, as expressed in Equation (8).

(8) $$v=\frac{\Delta h}{\Delta t}$$

where *v* denotes deposition rate, Δ*h* represents deposit thickness and Δ*t* corresponds to deposition time. At a scanning speed of 10 mm/s, the deposition rate of USJS-ECD attains 1.72 μm/min, which is approximately 13.91% greater than that observed for UAS ECD, measured at 1.51 μm/min (see Figure 5b). Nevertheless, for USJS-ECD, both the deposition thickness and deposition rate demonstrate a declining trend as the scanning speed increases. This phenomenon can be attributed to two primary factors: firstly, a lower scanning speed extends the residence time of the high-energy active deposition zone projected by the slit onto the cathode surface, thereby maintaining relatively stable interfacial reaction conditions and promoting the continuous reduction of nickel ions [25]. Secondly, at elevated scanning speeds, the synergistic effect of planar jet impingement combined with rapid scanning motion enhances interfacial renewal [26], which shortens the effective deposition time per unit area and consequently diminishes the deposition rate. Notably, at the minimal scanning speed of 0.1 mm/s, the deposition rate reaches 33.2 nm/s (equivalent to 1.99 μm/min), closely aligning with the theoretically calculated vertical deposition rate of 34 nm/s. This correspondence suggests that USJS-ECD approaches a quasi-steady-state deposition regime under low-velocity conditions. Through the combined effects of jet-enhanced mass transfer, continuous nickel ion supply via anodic dissolution, and localized electric field concentration, USJS-ECD achieves a superior deposition rate compared to UAS-ECD under identical experimental parameters.

### 4.2. Microstructure of Nickel Samples

**Figure 6:**
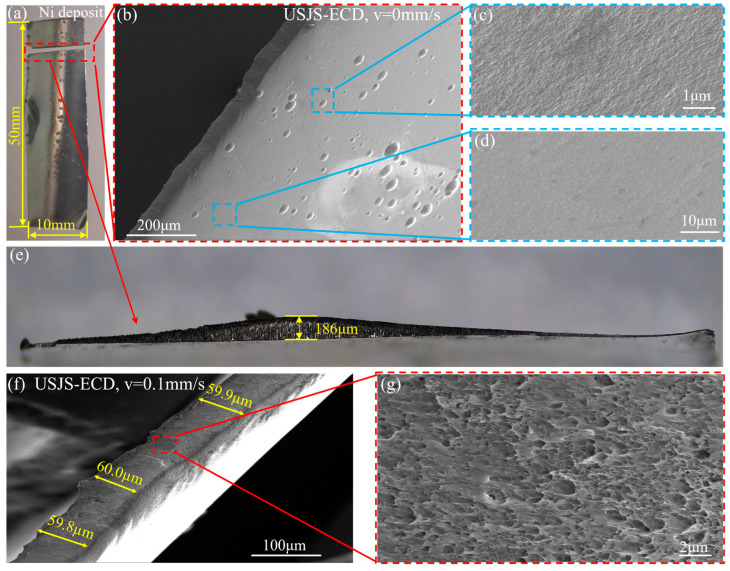
Photographs and SEM images of nickel samples. (**a**) Photograph of the nickel deposit (10 mm in width and 50 mm in length) formed after 30 min with fixed anode chamber; (**b**–**d**) SEM images of different regions of the nickel sample prepared under the fixed anode chamber condition; (**e**) cross sectional image of the deposit corresponding to (**a**); (**f**) cross sectional SEM image of the nickel sample prepared by USJS-ECD; (**g**) enlarged SEM image of the cross section shown in (**f**).

Nickel deposits fabricated via USJS-ECD at a scanning speed of 0 mm/s are depicted in Figure 6a–e. Notably, substantial deposition occurs within an effective width of approximately 10 mm, with the central region attaining a thickness of roughly 186 μm. This thickness rapidly diminishes toward the lateral edges, resulting in a distinctly bulged thickness profile centered along the deposit. The experimentally observed deposition morphology and thickness distribution are generally consistent with the stationary simulation results, particularly in terms of localized deposition behavior and central thickness accumulation. However, under static deposition conditions, as the deposit grows, the tip effect becomes increasingly significant, leading to electric field concentration in the central region and a corresponding increase in local current density, which in turn accelerates the deposition rate. In contrast, the simulation model is based on idealized assumptions and does not fully capture this electric field concentration effect. Consequently, the simulated deposition rate is underestimated, with the experimentally measured static deposition rate being approximately three times higher than the simulated value. Nevertheless, the surface morphology is predominantly characterized by nodular protrusions and an overall roughened texture. These experimental observations further indicate that, under stationary conditions, the electric field is highly localized within the jet impingement zone, resulting in pronounced current-density concentration at the central region of the deposit.

Upon implementing planar jet scanning, for instance at a speed of 0.1 mm/s, the deposition behavior undergoes a fundamental transformation. As illustrated in Figure 6f, the scanning motion induces a significant temporal averaging effect on the localized electric field along the lateral axis. Consequently, the initially concentrated current density peaks are effectively mitigated, facilitating the formation of a macroscopically uniform nickel deposit. Cross-sectional SEM analyses presented in Figure 6g demonstrate relatively minor thickness variations and low overall porosity, with only sporadic micropores or shallow pits observed in localized areas. These microstructural features are likely attributable to microscale flow disturbances or minor side reactions involving hydrogen evolution [27].

**Figure 7:**
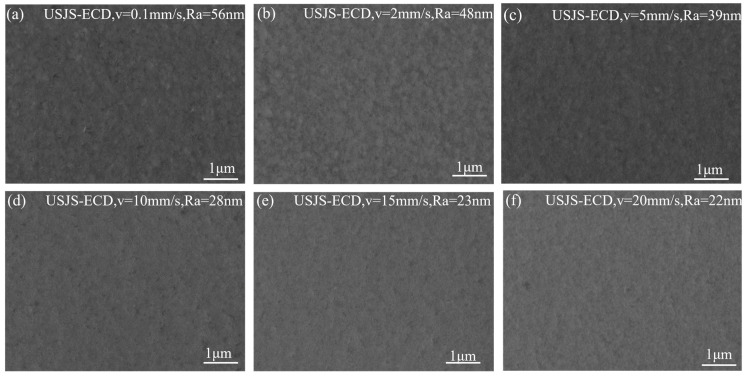
SEM images and surface roughness of nickel samples obtained by USJS-ECD at scanning speeds of (**a**) 0.1 mm/s; (**b**) 2 mm/s; (**c**) 5 mm/s; (**d**) 10 mm/s; (**e**) 15 mm/s and (**f**) 20 mm/s.

Ultranarrow-slit jet scanning (USJS) effectively mitigates nodular morphological instability caused by current concentration under stationary conditions and significantly enhances the surface quality of the resulting deposits. Surface scanning electron microscopy (SEM) images are presented in Figure 7 to illustrate these effects. Under scanning conditions, nickel deposits produced via USJS-ECD demonstrate notable planarization and densification. As the scanning speed increases from 0.1 mm/s to 20 mm/s, the surface roughness (Ra) progressively decreases from approximately 56 nm to around 22 nm, representing an overall reduction of approximately 60.71%. Within the scanning speed range of 10 to 20 mm/s, the surface roughness of USJS-ECD coatings remains consistently low, between 22 and 25 nm, while the surface morphology maintains continuity and compactness without exhibiting instability associated with excessive scanning speeds.

**Figure 8:**
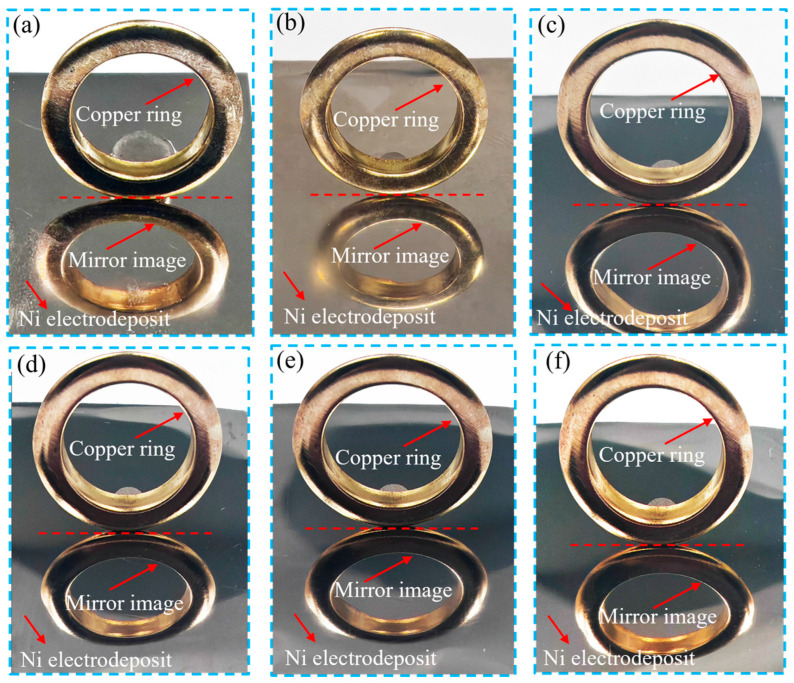
Photographs of nickel samples fabricated by USJS-ECD at different scanning speeds: (**a**) 0.1 mm/s; (**b**) 2 mm/s; (**c**) 5 mm/s; (**d**) 10 mm/s; (**e**) 15 mm/s and (**f**) 20 mm/s.

**Figure 9:**
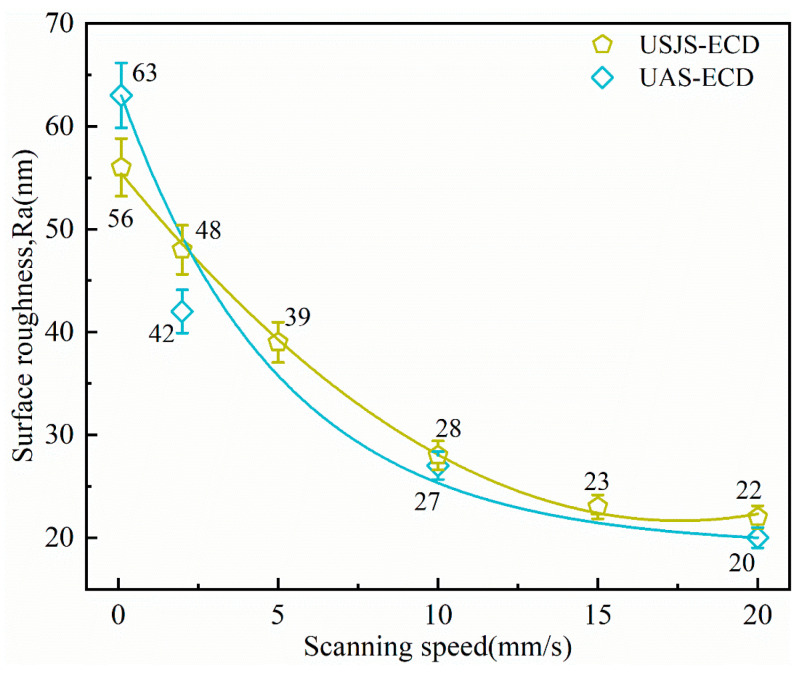
Comparison of surface roughness (Ra) of nickel deposits prepared by USJS-ECD and UAS-ECD [24].

The improvement in microscopic surface morphology is also clearly reflected at the macroscopic scale. As shown in Figure 8a–f, the surface reflectivity of the nickel deposits is progressively enhanced with increasing scanning speed. Under low-speed conditions (0.1–5 mm/s), the reflected contours appear relatively blurred and the overall surface uniformity is poor. In contrast, when the scanning speed is increased to 10–20 mm/s, the reflected contours become well-defined, exhibiting a pronounced mirror-like reflectivity with uniform surface gloss. A Further comparison between the USJS-ECD and UAS-ECD processes (Figure 9) reveals that the overall surface roughness (Ra) of the nickel deposits obtained by both methods is at a comparable level. However, within the higher scanning speed range (10–20 mm/s), the Ra values of the USJS-ECD samples are slightly higher than those of the UAS-ECD counterparts by approximately 3.5–9.1%. This difference is consistent with the grain size evolution discussed in the subsequent section, where a higher degree of grain refinement achieved under UAS-ECD conditions contributes to a further reduction in surface roughness. Despite this, the nickel deposits fabricated by USJS-ECD are capable of maintaining low surface roughness and a dense surface morphology over a relatively wide processing window. No evident surface defects or morphological instabilities are observed, indicating favorable deposition stability and good process adaptability.

**Figure 10:**
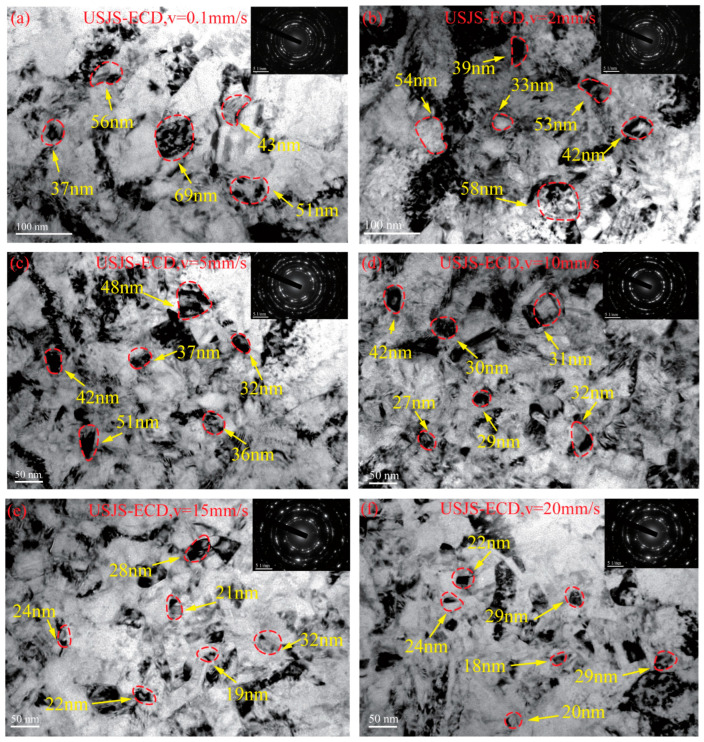
TEM images of nickel deposits obtained by USJS-ECD at different scanning speeds: (**a**) 0.1 mm/s; (**b**) 2 mm/s; (**c**) 5 mm/s; (**d**) 10 mm/s; (**e**) 15 mm/s and (**f**) 20 mm/s.

Bright-field transmission electron microscopy (TEM) images of nickel deposits synthesized at different scanning speeds are shown in Figure 10. As evident from panels 10a through 10f, the grain size of the nickel samples demonstrates a general decreasing trend with increasing scanning speed. Specifically, within the lower scanning speed range of 0.1 to 5 mm/s, the deposits predominantly consist of relatively coarse grains, approximately 38 to 70 nm in size, accompanied by a smaller proportion of finer grains. Conversely, at higher scanning speeds between 10 and 20 mm/s, a marked grain refinement is observed, with the grain size distribution becoming more uniform and primarily spanning 10 to 35 nm. Concurrently, selected area electron diffraction (SAED) patterns progressively transition toward more continuous diffraction rings, corroborating the refinement of the grain structure.

Furthermore, the grains across all samples predominantly exhibit polygonal morphologies rather than the typical equiaxed chain-like structures [28]. Integrating these observations with prior simulation results reveals the formation of a strong potential gradient concentration region near the slit outlet, which induces a high local overpotential at the electrode interface. Simultaneously, the sustained supply of Ni^2+^ ions enhance interfacial mass transfer, facilitating nearly simultaneous nucleation of grains under conditions of high nucleation density. Consequently, rapid impingement among adjacent nuclei restricts their growth space, promoting cooperative deformation and ultimately resulting in the formation of a dense, irregular polygonal nanocrystalline structure.

**Figure 11:**
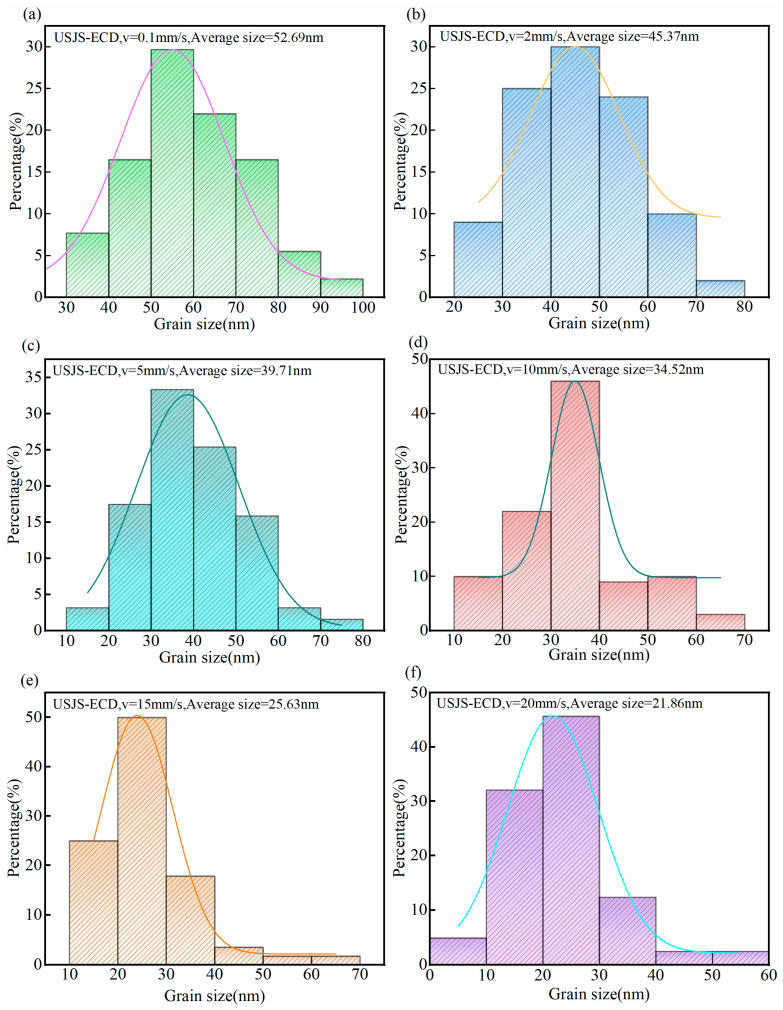
Grain size statistics of nickel samples obtained by USJS-ECD at different scanning speeds. (**a**) v = 0.1 mm/s, average size = 52.69 nm; (**b**) v = 2 mm/s, average size = 45.37 nm; (**c**) v = 5 mm/s, average size = 39.71 nm; (**d**) v = 10 mm/s, average size = 34.52 nm; (**e**) v = 15 mm/s, average size = 25.63 nm; (**f**) v = 20 mm/s, average size = 21.86 nm.

To corroborate the qualitative observations obtained via transmission electron microscopy (TEM) and to acquire statistically representative data on the microstructural scale, a systematic analysis of grain size was performed on nickel deposits fabricated by USJS-ECD at varying scanning speeds, as illustrated in Figure 11. Specifically, TEM samples were collected from three distinct locations (center, left, and right regions) of the deposited layer under each condition to minimize the influence of local microstructural heterogeneity and ensure spatial representativeness. Furthermore, grain size statistics were obtained from at least 100 randomly selected grains measured from multiple bright-field TEM micrographs for each sample, thereby ensuring statistically representative results. The results demonstrate a pronounced and consistent refinement of grain size with increasing scanning speed. Concurrently, the grain size distribution transitions from a broad, right-skewed profile to a more narrowly concentrated unimodal distribution. Specifically, as the scanning speed increases from 0.1 mm/s to 20 mm/s, the average grain size decreases progressively from 52.69 nm to 45.37 nm, 39.71 nm, 34.52 nm, 25.63 nm and ultimately 21.86 nm. At elevated scanning speeds (15–20 mm/s), the grain size distribution exhibits a marked sharpening with substantially reduced variability, indicative of enhanced microstructural uniformity.

**Figure 12:**
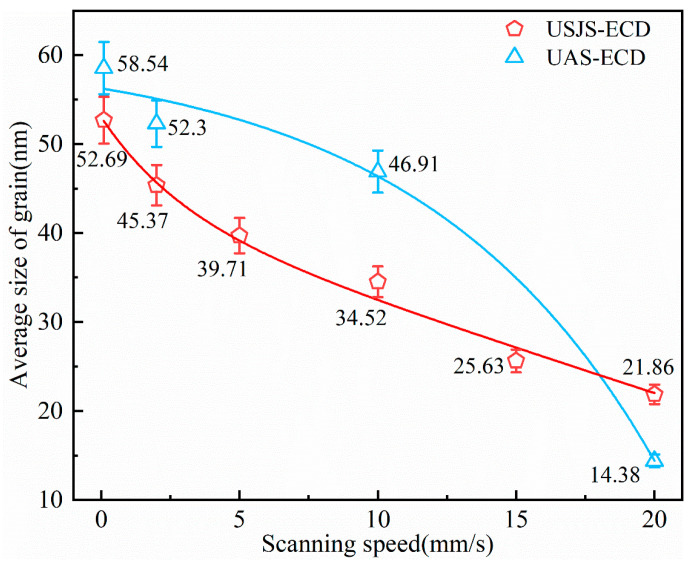
Comparison of average grain size between USJS-ECD and UAS-ECD [24] at different scanning speeds.

A comparison of the average grain size between USJS-ECD and UAS-ECD across various scanning speeds is shown in Figure 12. Within the scanning speed interval of 0.1 to 15 mm/s, USJS-ECD demonstrates a pronounced superiority in grain refinement relative to UAS-ECD. Conversely, at an elevated scanning speed of 20 mm/s, the grain refinement efficacy of USJS-ECD diminishes, becoming approximately 37.46% less effective than that of UAS-ECD. Despite this reduction in refinement capability at higher velocities, USJS-ECD maintains a more consistent and controllable refinement trend within the low to intermediate speed range. The grain size exhibits a well-defined gradient evolution as scanning speed increases. In contrast, UAS-ECD displays greater variability in grain size, characterized by more abrupt fluctuations. These findings suggest that USJS-ECD offers enhanced stability and controllability in grain size regulation, facilitating smooth refinement through improved mass transfer uniformity and increased interfacial growth stability [29].

**Figure 13:**
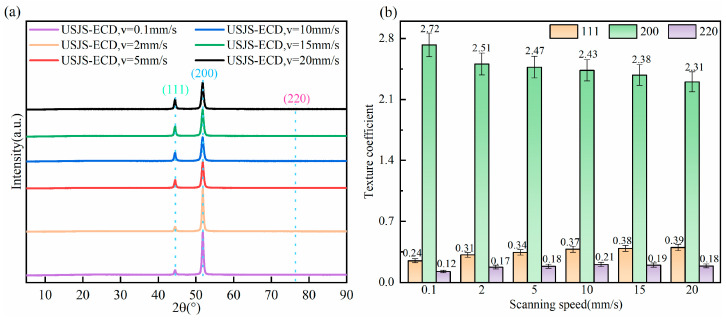
(**a**) XRD patterns of electrodeposited nickel under different scanning speeds. (**b**) Texture coefficients of different crystal planes at various scanning speeds.

X-ray diffraction (XRD) patterns of nickel deposits synthesized by USJS-ECD at different scanning speeds are shown in Figure 13a. All samples exhibit characteristic diffraction peaks corresponding to face-centered cubic (FCC) nickel, with primary reflections located at approximately 2θ values of 44.5°, 51.8° and 76.4°, which correspond to the (111), (200) and (220) crystallographic planes, respectively. The absence of additional diffraction peaks indicates that the phase composition remained unchanged under all experimental conditions. With increasing scanning speed from 0.1 to 20 mm/s, systematic variations in the relative intensities of the diffraction peaks were observed. Specifically, the intensity of the (111) peak gradually increased, whereas that of the (200) peak decreased correspondingly. Combined with the texture coefficient (the calculation method for texture coefficient is provided in the Supplementary Materials S1) analysis shown in Figure 13b, it can be seen that only the texture coefficient of the (200) plane remained greater than 1, while those of the (111) and (220) planes were consistently lower than 1, indicating that the deposits still exhibited a preferred (200) orientation. Nevertheless, although the (111) plane was not the dominant preferred orientation, its contribution to the microstructural evolution should not be neglected. Owing to its densely packed atomic arrangement and relatively low surface energy, the enhancement of the (111) orientation reduces the probability of interfacial defect formation and promotes the densification of the deposited nickel layer [30]. This interpretation is consistent with the SEM observations discussed previously, where smoother and denser surface morphologies were obtained at higher scanning speeds.

In contrast, the preferentially oriented (200) plane is generally associated with a columnar growth tendency and relatively coarser grain features in electrodeposited nickel. The dominance of this orientation contributes to improved tensile strength by enhancing crystallographic anisotropy and load-bearing capability along the growth direction. However, the accompanying increase in effective grain size may weaken the grain boundary strengthening effect and reduce the resistance to localized plastic deformation, which is considered one of the contributing factors to the decrease in microhardness observed at higher scanning speeds [31]. Taken together, these results suggest that planar jet scanning electrodeposition not only regulates the deposition rate but also significantly influences the crystallographic orientation selection during film growth.

### 4.3. Mechanical Properties

**Figure 14:**
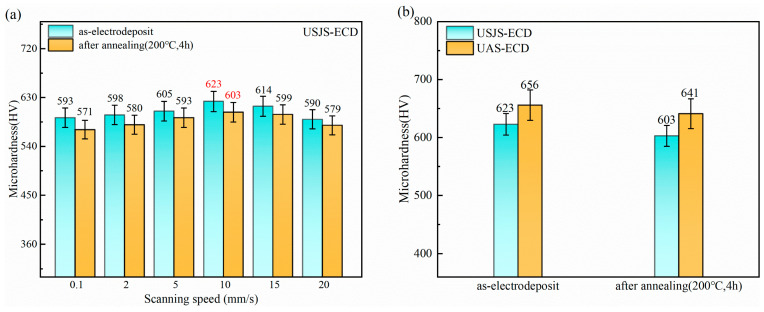
(**a**) Microhardness of nickel deposits prepared by USJS-ECD at different scanning speeds in the as-electrodeposited state and after annealing at 200 °C for 4 h; (**b**) comparison of maximum microhardness between USJS-ECD and UAS-ECD [24] deposit in the as-electrodeposited state and after annealing.

The hardness of nickel samples prepared by USJS-ECD at different scanning speeds is presented in Figure 14a. The nickel layer produced by USJS-ECD at a scanning velocity of 10 mm/s demonstrates a relatively high hardness value of 623 HV, which is marginally lower than that obtained by UAS-ECD (656 HV), as show in Figure 14b. This discrepancy is primarily attributed to differences in material purity and microstructural characteristics. In the USJS-ECD process, a nickel anode with a purity of ≥99.9% serves as the active electrode. During deposition, anodic dissolution inevitably introduces trace impurities such as sulfur, nitrogen, carbon, oxygen, chlorine, iron, and copper into the electrolyte, with their concentrations detailed in Table 2. Although these impurities are present at low levels, they may be incorporated into the deposited layer as solid solutions or inclusions, thereby influencing grain boundary structures and dislocation mobility, which in turn moderately reduces microhardness [12].

Furthermore, annealing the as-deposited nickel samples at 200 °C for 4 h results in a slight decrease in microhardness; however, this reduction remains limited. This behavior is mainly ascribed to the (111) crystallographic orientation of the deposited layer, as previously discussed. This orientation is associated with lower surface energy and a more stable atomic packing arrangement, which constrains structural evolution during heat treatment and mitigates significant softening. Although the hardness of the USJS-ECD samples is somewhat lower than that of UAS-ECD, it remains comparatively high. Taken together with prior findings, this method effectively balances a substantially increased deposition rate, enhanced surface densification, and relatively stable mechanical properties.

**Figure 15:**
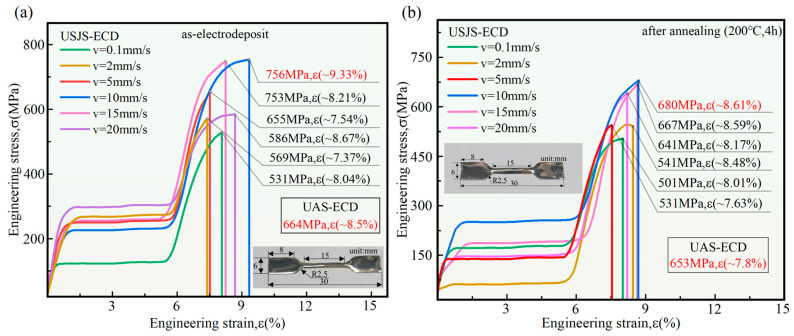
Engineering stress-engineering strain relationship. (**a**) as-electrodeposit nickel; (**b**) annealed nickel.

Tensile mechanical behavior of nickel deposits fabricated under varying conditions is shown in Figure 15. Both the as-electrodeposited and annealing-treated samples were tested with the same sample dimensions shown in the inset of Figure 15. The as-electrodeposited sample produced at a scanning speed of 10 mm/s demonstrates notably superior overall mechanical performance, exhibiting an tensile strength of approximately 756 MPa and an elongation to fracture of around 9.33%. In comparison, the UAS-ECD sample prepared under identical scanning conditions shows a tensile strength of 664 MPa and elongation of 8.5%, representing improvements of approximately 13.85% in strength and 9.76% in ductility for the USJS-ECD sample (see Figure 15a). This highlights the significant advantage of USJS-ECD in synergistically optimizing both strength and plasticity.

Following annealing at 200 °C for 4 h (see Figure 15b), both tensile strength and elongation to fracture decrease to varying extents. Despite a certain reduction in strength observed in the USJS-ECD samples relative to UAS-ECD, the optimal tensile strength remains approximately 680 MPa, with elongation to fracture reaching about 8.61%. These values surpass the corresponding optimal metrics for UAS-ECD, which are 653 MPa and 7.8%, respectively.

Moreover, while achieving an excellent balance between strength and ductility, the USJS-ECD samples exhibit a relatively gradual increase in tensile stress within the intermediate region of the stress-strain curve. This behavior is attributed to the combined effects of multiple factors. Firstly, as previously noted, impurity elements may modulate the material’s deformation behavior by affecting dislocation motion and grain boundary structures [32]. Secondly, relatively high internal stresses are inherently introduced during electrodeposition, arising from grain refinement, nonequilibrium growth, and the accumulation of dislocation structures [33].

**Figure 16:**
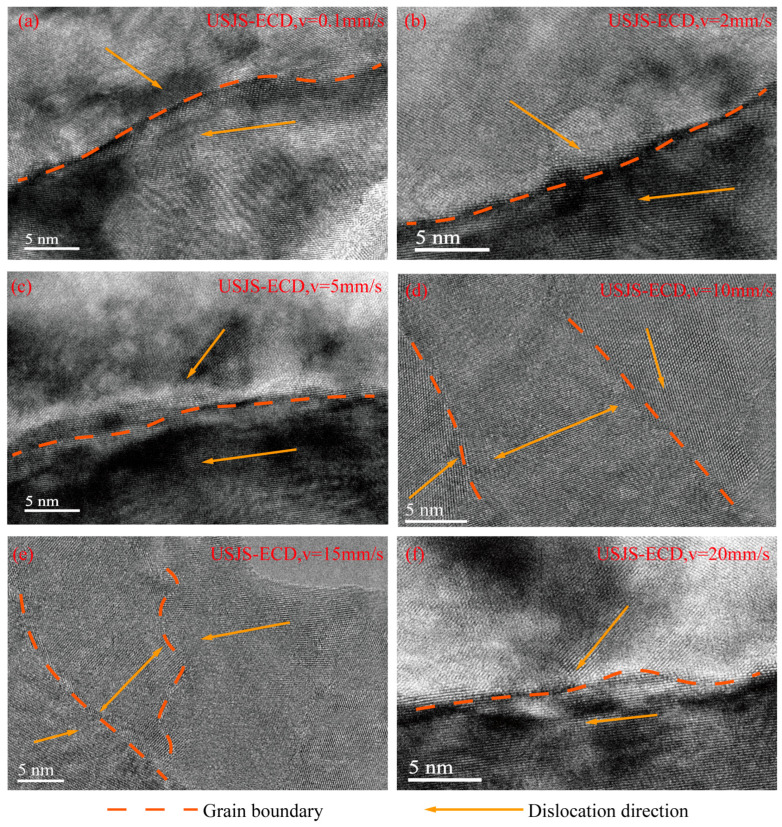
TEM images of dislocation structures in nickel samples prepared by USJS-ECD under different scanning speeds: (**a**) 0.1 mm/s; (**b**) 2 mm/s; (**c**) 5 mm/s; (**d**) 10 mm/s; (**e**) 15 mm/s; (**f**) 20 mm/s.

**Figure 17:**
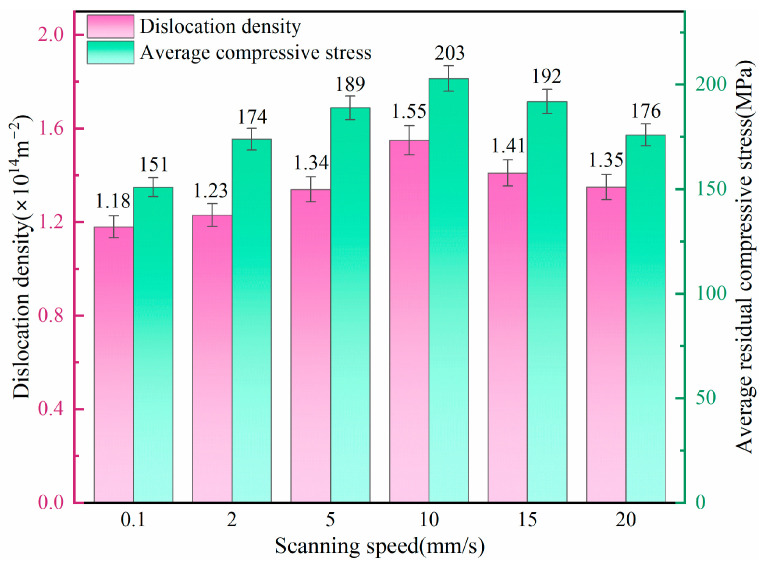
Variations in dislocation density and average residual compressive stress of nickel deposits fabricated by USJS-ECD under different scanning speeds.

Dislocation structures distributed along grain boundaries were observed in the nickel deposits fabricated at different scanning speeds, with noticeable variations in their orientations and distribution characteristics, as shown in Figure 16a–f. Owing to the incomplete annihilation of dislocations with different orientations during deposition, residual internal stresses were inevitably accumulated within the deposits. The variations in dislocation density and residual compressive stress with scanning speed are presented in Figure 17 (the calculation method for dislocation density is provided in the Supplementary Materials S2). It was found that both the dislocation density and residual compressive stress initially increased and subsequently decreased with increasing scanning speed, which was consistent with the evolution trend of the tensile strength. At a scanning speed of 10 mm/s, the maximum values were obtained, reaching 1.55 × 10^14^ m^−2^ for the dislocation density and 203 MPa for the residual compressive stress. The relatively high dislocation density indicates the presence of enhanced lattice distortion and defect storage capability within the deposits, which increases the resistance to dislocation motion and thereby contributes to the improvement of both yield strength and ultimate tensile strength [34].

Meanwhile, the residual compressive stress generated during deposition imposes a constraining effect on plastic deformation, further strengthening the mechanical response of the material. During tensile deformation, the externally applied stress gradually promotes the partial release of the residual internal stress, resulting in a characteristic intermediate stage in which the strain continues to increase while the stress develops in a relatively uniform manner [35]. The associated stress relaxation and dislocation rearrangement processes are considered beneficial for delaying localized plastic instability, thereby contributing to the simultaneous enhancement of strength and ductility.

It is important to note that annealing promotes partial relaxation of internal stress and rearrangement of dislocation structures, thereby causing a slight reduction in strength while rendering the mechanical response more stable and more reflective of intrinsic material properties. In summary, the USJS-ECD process achieves a coordinated improvement in strength and ductility through the regulation of grain structure and defect states, albeit with the inevitable introduction of a certain level of internal stress. Appropriate heat treatment facilitates an optimal balance between mechanical strength and structural stability.

**Figure 18:**
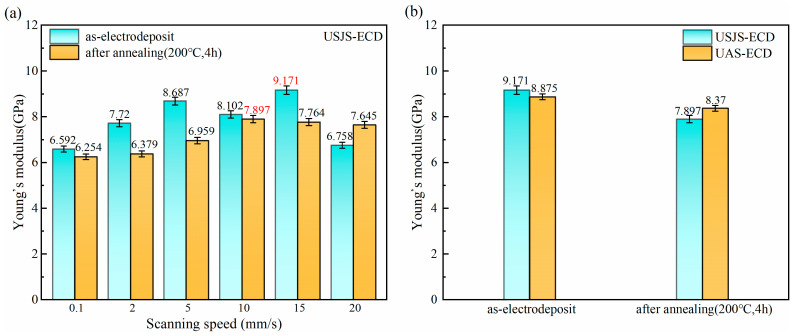
(**a**) Variation in Young’s modulus of nickel samples prepared by USJS-ECD at different scanning speeds; (**b**) comparison of maximum Young ’s modulus of samples prepared by USJS-ECD and UAS-ECD [24].

To further evaluate the tensile deformation behavior of nickel deposits fabricated by USJS-ECD, the apparent Young ’s modulus was estimated from the initial linear region of the engineering stress–strain curves, as shown in Figure 18a. The Young’s modulus derived from the tensile stress–strain curves represents an apparent modulus affected by system compliance. Additional nanoindentation measurements yielded Young ’s modulus values of 182–194 GPa, which are within the range commonly reported for nickel, indicating that the low tensile-derived values do not reflect the intrinsic elastic properties of the deposits. However, because both USJS-ECD and UAS-ECD specimens were evaluated under identical testing conditions, the apparent modulus values remain useful for assessing the relative differences in tensile deformation behavior between the two processes. Within this context, the apparent Young ’s modulus of the as-electrodeposited USJS-ECD nickel samples exhibited a non-monotonic dependence on scanning speed, characterized by an initial increase followed by a decrease with partial recovery. At a scanning speed of 15 mm/s, the apparent modulus reached a maximum value of approximately 9.171 GPa, slightly exceeding the value of 8.875 GPa obtained for UAS-ECD (Figure 18b). After annealing, however, the corresponding value became approximately 5.65% lower than that of UAS-ECD.

The relative variation in apparent stiffness with scanning speed is considered to be associated with the evolution of microstructural features, including grain morphology, defect density, and residual stress state. In the as-electrodeposited condition, the presence of high residual internal stress and unrecovered dislocation structures may contribute to a relatively stronger resistance to initial elastic-plastic deformation. Following annealing, partial stress relaxation and dislocation rearrangement reduce this constraint effect, leading to a more stable mechanical response and improved deformation uniformity. In summary, although the absolute modulus values are influenced by experimental compliance effects, the comparative results still suggest that the USJS-ECD process significantly affects the tensile deformation behavior and structural integrity of the nickel deposits through microstructural regulation and defect-state evolution [36].

## 5. Conclusions

To attain nanocrystalline nickel characterized by high hardness, enhanced toughness, and elevated purity, while simultaneously preserving deposition efficiency and cost-effectiveness, ultranarrow slit-jet scanning electrodeposition (USJS-ECD) technique is proposed. This method incorporates a narrow slit with an extended-span jet executing reciprocating motion in close proximity to the cathode, thereby introducing synergistic effects of forced convection and dynamically localized electric fields. Compared to UAS-ECD, this approach markedly improves deposition efficiency, microstructural uniformity, and the balance between hardness and toughness. The principal findings are summarized as follows:

(1) The USJS-ECD process facilitates the integration of high purity, refined nanocrystalline microstructure, superior strength-toughness synergy, and a stable deposition rate through the combined influence of planar jet scanning and localized electric fields. Under identical operational parameters, the deposition rate is enhanced by 13.91% relative to UAS-ECD. The favorable process controllability and scalability render this method a promising avenue for the fabrication of high-performance nanocrystalline metal foils.
(2) The periodic migration of localized electric fields is achieved via the narrow slit planar jet flow coupled with scanning motion, resulting in continuous renewal of mass transport conditions and electric field distribution at the deposition interface. This promotes high-density nucleation and uniform grain growth. The deposited nickel exhibits a typical face-centered cubic crystal structure with a purity of approximately 98.82 wt%. The average grain size ranges from 21.86 to 52.69 nm, accompanied by a stable (200) preferred orientation, demonstrating effective control over nanocrystalline structure and texture within high-purity systems.
(3) In the absence of additives, the nickel layer produced by USJS-ECD displays a surface roughness (Ra) of approximately 22 nm, microhardness around 623 HV, tensile strength of 756 MPa, and elongation at fracture of 9.33%. Compared to UAS-ECD, the hardness experiences a slight decrease, whereas tensile strength increases by 13.85%, indicating a commendable combination of mechanical properties characterized by both high hardness and toughness.

## Figures and Tables

**Table 1:** Simulation parameters.

Boundary	Parameter	Value
	Electrical conductivity	20 S/m
	Dynamic viscosity	0.0012 Pa·s
	Ni^2+^ concentration	1.4 mol/L
	Anodic charge transfer coefficient	1.5
	Diffusion coefficient of Ni^2+^	7 × 10^−10^ m^2^/s
	Electrolyte density	1400 kg/m^3^
1	Electrolyte inlet	16 L/min
2	Anode voltage	20 V
3	Slit to cathode gap	200 μm
4	Cathode substrate voltage	0 V
	Ultranarrow slit opening width	50 μm

**Table 2:** Elemental composition of chemical constituents in nickel samples prepared by USJS-ECD.

Element	Content (wt%)	Element	Content (wt%)
Ni	98.82	Fe	0.197
N	0.183	S	0.03
C	0.098	Cu	0.169
Cl	0.17	O	0.31

## Data Availability

The original contributions presented in the study are included in the article, further inquiries can be directed to the corresponding author.

## Supplementary Materials

The following supporting information can be downloaded at: https://www.mdpi.com/article/10.3390/mi17060700/s1. References [37,38] are cited in the supplementary materials.
