# Supplementary material for: Efficient Preparation of Ultrahigh-Strength Nanostructured Nickel by Ultranarrow Slit-Jet Scanning Electrodeposition Without Additives
Source: Micromachines (Basel). 2026 Jun 8;17(6):700. doi: 10.3390/mi17060700 (PMC13302857; doi:10.3390/mi17060700)
Supplement: Supplementary file 1 [file micromachines-17-00700-s001.zip › micromachines-4314669-supplementary.pdf]

micromachines

MDPI

Supplementary Materials

Supplementary Materials S1. Details of the Formula for Calculating Texture Coefficient

The crystallographic texture of the nickel deposits was further evaluated using the texture coefficient (TC), which was calculated according to the following equation [1]:

$$TC(hkl)=\frac{\dfrac{I(hkl)}{I_0(hkl)}}{\dfrac{1}{n}\sum_{i=1}^{n}\dfrac{I(hkl)_i}{I_0(hkl)_i}} \quad \text{(S1)}$$

where $I(hkl)$ is the measured diffraction intensity of the (hkl) plane, $I_0(hkl)$)is the standard diffraction intensity obtained from the PDF reference card(for the nickel PDF reference card, the standard diffraction intensity values $I_0(hkl)$ for the (111), (200), and (220) crystal planes are 100, 42, and 21, respectively), and n is the number of diffraction peaks used for calculation.

Supplementary Materials S2. Details of the Formula for Calculating Dislocation Density

The dislocation density (ρ) of the nickel deposits was evaluated based on the Williamson-Hall (W-H) analysis of the XRD peak broadening. The W-H equation is expressed as follows [2]:

$$\beta cos\theta = \frac{k\lambda}{D} + 4\epsilon sin\theta \quad \text{(S2)}$$

where β is the full width at half maximum (FWHM) of the diffraction peak (in radians), θ is the diffraction angle, k is the shape factor (taken as 0.9), λ is the X-ray wavelength (0.15406 nm for Cu Kα radiation), D is the crystallite size, and ϵ is the microstrain.

Subsequently, the dislocation density (ρ) was calculated using the following relation:

$$\rho = \frac{2\sqrt{3}\epsilon}{D \cdot b} \quad \text{(S3)}$$

where b represents the magnitude of the Burgers vector (0.249 nm for the present Ni electrodeposition layer).

References

1. Harris, G.B. X. Quantitative Measurement of Preferred Orientation in Rolled Uranium Bars. *Lond. Edinb. Dublin Philos. Mag. J. Sci.* **1952**, *43*, 113–123. https://doi.org/10.1080/14786440108520972.
2. Williamson, G.K.; Smallman, R.E., III. Dislocation Densities in Some Annealed and Cold-Worked Metals from Measurements on the X-Ray Debye-Scherrer Spectrum. *Philos. Mag. A J. Theor. Exp. Appl. Phys.* **1956**, *1*, 34–46. https://doi.org/10.1080/14786435608238074.

Disclaimer/Publisher's Note: The statements, opinions and data contained in all publications are solely those of the individual author(s) and contributor(s) and not of MDPI and/or the editor(s). MDPI and/or the editor(s) disclaim responsibility for any injury to people or property resulting from any ideas, methods, instructions or products referred to in the content.
